# Injectable and compression-resistant low-viscosity polymer/ceramic composite carriers for rhBMP-2 in a rabbit model of posterolateral fusion: a pilot study

**DOI:** 10.1186/s13018-017-0613-0

**Published:** 2017-07-11

**Authors:** Stefanie M. Shiels, Anne D. Talley, Madison A. P. McGough, Katarzyna J. Zienkiewicz, Kerem Kalpakci, Daniel Shimko, Scott A. Guelcher, Joseph C. Wenke

**Affiliations:** 1 US Army Institute of Surgical Research, Fort Sam Houston, TX USA; 2 Department of Chemical and Biomolecular Engineering, Vanderbilt University, Nashville, TN USA; 3 Department of Biomedical Engineering, Vanderbilt University, Nashville, TN USA; 4 Medtronic Spine LLC, Memphis, TN USA; 5 Center for Bone Biology, Vanderbilt University Medical Center, Nashville, TN USA

**Keywords:** Posterolateral fusion, Rabbit, Bone morphogenetic protein, Polyurethane, Injectable, Compression resistant

## Abstract

**Background:**

The challenging biological and mechanical environment of posterolateral fusion (PLF) requires a carrier that spans the transverse processes and resists the compressive forces of the posterior musculature. The less traumatic posterolateral approach enabled by minimally invasive surgical techniques has prompted investigations into alternative rhBMP-2 carriers that are injectable, settable, and compression-resistant. In this pilot study, we investigated injectable low-viscosity (LV) polymer/composite bone grafts as compression-resistant carriers for rhBMP-2 in a single-level rabbit PLF model.

**Methods:**

LV grafts were augmented with ceramic microparticles: (1) hydrolytically degradable bioactive glass (BG), or (2) cell-degradable 85% β-tricalcium phosphate/15% hydroxyapatite (CM). Material properties, such as pore size, viscosity, working time, and bulk modulus upon curing, were measured for each LV polymer/ceramic material. An in vivo model of posterolateral fusion in a rabbit was used to assess the grafts’ capability to encourage spinal fusion.

**Results:**

These materials maintained a working time between 9.6 and 10.3 min, with a final bulk modulus between 1.2 and 3.1 MPa. The LV polymer/composite bone grafts released 55% of their rhBMP-2 over a 14-day period. As assessed by manual palpation in vivo, fusion was achieved in all (*n* = 3) animals treated with LV/BG or LV/CM carriers incorporating 430 μg rhBMP-2/ml. Images of μCT and histological sections revealed evidence of bone fusion near the transverse processes.

**Conclusion:**

This study highlights the potential of LV grafts as injectable and compression-resistant rhBMP-2 carriers for posterolateral spinal fusion.

## Background

Spinal fusion surgeries are performed for a variety of ailments, such as disc degeneration, spinal stenosis, and degenerative spondylolisthesis, as well as traumatic injuries [1]. Autograft bone is the current gold standard due to its osteoconductive, osteoinductive, and osteogenic properties. Traditional harvesting sights include the iliac crest and local bone following spinal decompression; however, these involve risk of donor site morbidity or inadequate bone supply.

Synthetic bone grafts have been investigated as alternatives to autograft. The growth factor recombinant human bone morphogenetic protein-2 (rhBMP-2) promotes differentiation, maturation, and proliferation of multipotent cells and thus is approved for clinical use for posterolateral lumbar spinal arthrodesis as either an autograft bone extender or bone graft substitute [2]. Current protocol involves delivery of rhBMP-2 from an absorbable collagen sponge (ACS) from within a compression-resistant metallic tapered spinal fusion cage (LT-CAGE™, Medtronic, Minneapolis, MN) for interbody fusion [3, 4]. Use of rhBMP-2-loaded ACS in a posterolateral fusion procedure is more challenging than interbody fusion due to the less favorable biological and mechanical conditions for bone healing [5]. Recent studies involving combining the rhBMP-2/ACS with a bulking agent, such as calcium phosphate granules, show improved posterolateral fusion rates in a non-human primate model compared to rhBMP-2/ACS alone, revealing the utility of a compression-resistant rhBMP2 carrier [5–10].

For placement of graft materials, surgical approaches involving large incisions are often used. However, there is no standard methodology for application of grafting materials using minimally invasive surgical techniques (MIS). A MIS spinal fusion approach could potentially decrease both muscle destruction and length of hospital stay. There is considerable interest in the development of new bone grafts that can be delivered by MIS techniques: injectable and, upon curing, are resistant to the compressive forces of the posterior musculature [10]. However, this combination of favorable properties cannot be achieved using currently available carriers.

The goal of this research project was to develop and evaluate the feasibility of a rhBMP-2-loaded ceramic composite. Here, injectable low-viscosity poly(ester urethane)(LV-PUR)/ceramic composite bone grafts are introduced in a rabbit model of posterolateral fusion (PLF). These LV grafts exhibit setting times of 5–10 min [11–13], support diffusion-controlled release of rhBMP-2, and promote healing in rodent, dog, and sheep models of bone regeneration [14–17].

## Methods

### Material preparation and characterization

The LV-PUR/ceramic composite grafts were prepared in a multistep process: (1) preparation of a polyester triol which is the foundation of the LV-PUR, (2) surface modification of the ceramic particles, and (3) combination of the LV-PUR, the ceramic component, and a polymerization catalyst to form a composite graft. Materials were purchased from Sigma-Aldrich (St. Louis, MO), Polysciences (Warrington, PA), or Ricerca Biosciences LLC (Painesville, OH), unless otherwise stated. The polyester triol was synthesized as described previously [18, 19]. The polyester backbone was composed of 70% ε-caprolactone, 20% glycolide, and 10% DL-lactide, with a molecular weight of 450 g mol^−1^.

LV-PUR/ceramic composite grafts were augmented with one of two types of ceramic particles to enhance handling properties and to achieve compression-resistant mechanical properties [16]: LV/BG contained 45S5 bioactive glass (BG) particles (150–212 μm, Mo-Sci Corporation, Rolla, MO) and the LV/CM contained 85/15 β-tricalcium phosphate /hydroxyapatite ceramic (CM) particles (MASTERGRAFT® Mini Granules, Medtronic Spinal and Biologics, Memphis, TN). The surfaces of the BG particles were modified as described previously [20–22], which grafted a ε-caprolactone onto the surface.

To create the LV-PUR/ceramic composite grafts (LV/BG and LV/CM), the components were mixed in a two-step method [16]. In the first step, the polyester triol, ceramic particles (either 45 wt% BG or 45 wt% CM), and triethylene diamine (TEDA) in dipropylene glycol (DPG) at 1.1 pphp were combined and mixed by hand for 30 s. Lysine-triisocyanate (LTI-PEG) prepolymer and lyophilized rhBMP-2 (430 μg rhBMP-2/mL cured graft; Medtronic, Memphis, TN) were added to the first step and mixed by hand for 60 s prior to rheology or placement into a wound. The loading of ceramic particles was 45 wt%, which was the highest possible concentration that maintained injectability [23].

#### Rheological analysis

To test the viscosity of the initial composites, triplicate samples were prepared without TEDA catalyst to prevent curing during testing. The polyester triol, LTI-PEG, and ceramic particles were mixed by hand for 60 s and poured between 40-mm cross-hatched parallel plates on a AR2000ex rheometer (TA Instruments, New Castle, DE). The plates were compressed to a gap of 1000 μm and subjected to a dynamic frequency sweep (0.5 to 100 rad s^−1^) at 25 °C with controlled strain amplitude of 0.02%. A Cox-Merz transformation was applied to the collected dynamic data to obtain the steady state viscosity (*η*, Pa s) as a function of shear rate (*γ*, s^−1^). The curing profile of the LV-PUR/ceramic composites was also determined using a rheometer. For these, the polyester triol, TEDA catalyst, and ceramic particles were mixed by hand initially for 30 s. The LTI-PEG prepolymer was added, and the samples were mixed by hand for 60 s. Triplicate reactive samples were loaded onto 25-mm disposable aluminum plates and compressed to a gap of 1000 μm. An oscillatory time sweep was run on triplicate samples with a frequency of 1 Hz and a controlled amplitude of 1% strain. The storage (G′) and loss (G″) moduli were collected over time. The working time was defined as the cross-over point of G′ and G″. The tack-free time (TFT) of LV-PUR/ceramic composite grafts were measured as the time at which the material no longer stuck to a metal spatula.

#### Porosity and pore size

Images of cured LV-PUR/ceramic composite specimens were acquired at a voltage of 1 kV using an S-4200 scanning electron microscope (Hitachi, Schaumberg, IL). Diameters of pores, both open and closed, were measured, using ImageJ 1.47p image analysis software, of three images for each treatment group. Porosity was determined gravimetrically [18].

#### Mechanical properties

Cylindrical samples (6 × 12 mm) of cured LV-PUR/ceramic composites were prepared in the presence of water to mimic in situ curing and tested under compression using an MTS 898 Bionix system (Eden Prairie, MN). Samples were submerged in phosphate-buffered saline for 24 h prior to testing, preloaded to 3 N, and compressed at a constant rate of 25 mm/min. Engineering stress was calculated from the original cross-sectional area of the cylinders. The compressive strength was reported as the stress measured at sample failure, and the bulk (compressive) modulus was calculated as the slope of the initial linear portion of the stress-strain curve. Bulk modulus and compressive strength are presented as mean ± standard deviation of triplicate samples.

#### rhBMP-2 release kinetics

LV/CM scaffolds (~50 mg, *n* = 3) loaded with rhBMP-2 (100 μg rhBMP-2/mL scaffold) were incubated in 1 mL α-Minimum Essential Medium with 1% bovine serum albumin at 37 °C [24]. Elution media was collected and replenished daily to minimize rhBMP-2 degradation. The concentration of rhBMP-2 was measured for 14 days using a human recombinant BMP-2 Quantikine ELISA kit (R&D systems, Minneapolis, MN).

### Rabbit model of posterolateral fusion: pilot study

All animal procedures were approved by the Institutional Animal Care and Use Committee of the US Army Institute of Surgical Research, Fort Sam Houston, TX, and were conducted in compliance with the Animal Welfare Act, the implementing Animal Welfare Regulations, and the principles of the Guide for the Care and Use of Laboratory Animals. Prior to surgery, the individual components of the LV-PUR/ceramic composite grafts were gamma-irradiated at a dose of 25 kGY to ensure sterility. Six skeletally mature rabbits (4.35 ± 0.49 kg) received a single level, bilateral, posterolateral fusion [25]. Approximately 30 min prior to surgery, a fentanyl patch (25 μg/kg) was affixed to a shaved portion of the rabbit’s dorsum and a pre-emptive dose of hydromorphone (0.15 mg/kg) was given. Anesthesia was induced with ketamine and xylazine (25 and 5 mg/kg) and maintained with isoflurane. A dorsal midline skin incision was made over the spinous processes from L4–L7 followed two paramedian fascial incisions. The longissimus muscles were retracted to expose the medial aspect of the transverse processes of L5 and L6. The transverse processes were decorticated with an electric burr. Following exposure, the LV-PUR/ceramic composite components were mixed along with the rhBMP-2 (430 μg/mL cured graft) and ceramic particles (CM or BG), packed into a syringe, and injected onto the site over the intertransverse ligament spanning the L5 and L6 transverse processes (Table 1). The injected composite expanded to a final volume of ~3 mL per fusion site after the reaction was complete. The incisions were approximated and the animals recovered following computed tomography (CT; Prime Aquilion TSX-303A, Toshiba, Tokyo, Japan). CT scans were performed immediately post-operative and at 4- and 8-week post-operative with slice thickness of 0.5 mm. The rabbits were anesthetized and euthanized after 8 weeks. The spinal segments from L4–L7 were excised and evaluated for fusion by manual palpation, plain radiography (Faxitron MX20), and CT by skilled outside observers, including an orthopedic surgical resident and clinical veterinarian. Motion of the L5–L6 segment was determined by gentle flexion and extension of the fusion site. A successful fusion was specified as cases where no movement at the intervertebral disc was present. The excised spines were fixed in 10% formalin prior to analysis by microcomputed tomography (μCT) and histology.

**Table 1:** In vivo study design and LV-PUR/ceramic composite characteristics

Treatment group	Ceramic component	Particle diameter (μm)	Ceramic composition (wt%)	Number
LV/BG	Bioglass (45S5)	150–212	45	3
LV/CM	85/15 β-tricalcium phosphate/hydroxyapatite ceramic	100–500	45	3

Data are shown as mean ± SD

#### μCT analysis

The excised spinal segments were cut along the sagittal plane to separate the left and right fusion bodies. A μCT50 (SCANCO Medical, Basserdorf, Switzerland) was used to acquire scans of the excised spines at 70 kVp energy, 200 μA source current, 1000 projections per rotation, 800 ms integration time, and an isotropic voxel size of 24.2 μm. The ossified tissue was segmented from soft tissue using the lower and upper threshold of 240 and 1000 mgHAcm^−3^, respectively, with a Gaussian noise filter settings of sigma 0.7 and support 2. Bone volume/total volume (BV/TV), trabecular number (TB.N.), trabecular thickness (Tb.Th.), and trabecular separation (Tb.Sp.) within the regions of interest were computed using SCANCO’s Medical μCT systems software as described previously [26].

#### Histology and histomorphometry

After fixation in formalin, the excised spinal halves were dehydrated in a graded series of ethanol and embedded in polymethylmethacrylate (PMMA). Using an Exakt band saw (Exakt Technologies, Oklahoma City, OK), transverse sections were cut. The sections were then ground and polished to <100 μm using an Exakt grinding system and stained with Sanderson’s rapid bone stain. New bone stained red, residual CM stained black, and infiltrating cells stained blue. Remaining BG particles appeared white and did not absorb the stain. Histological sections were used to visualize the amount of new bone formed in the fusion body and the residual CM and BG particles. MetaMorph® software (Molecular Devices, Sunnyvale, CA) was used to quantify the area of the bone in the fusion mass. The color threshold was set to account for the red stain (new bone), and the threshold settings were identical for all images. The area of interest was selected to include the fusion mass only and excluded regions outside the plane of the lateral processes.

### Statistical analysis

Data are plotted as mean ± standard deviation. Comparisons between groups were performed using unpaired *t* tests in GraphPad Prism (Graph Pad, La Jolla, CA). The significance level was defined as *p* < 0.05.

## Results

### Material properties

**Fig. 1:**
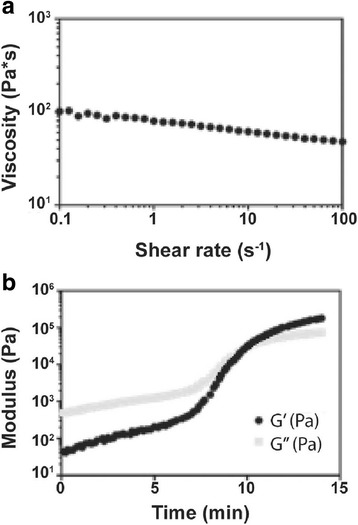
**a** Representative plot of viscosity versus shear for uncatalyzed (i.e., non-reactive) mixture showing shear-thinning properties. **b** Storage (G′) and loss (G″) moduli measured for the catalyzed (i.e., reactive) mixture. Data collection was started 2.5 min after mixing. The working time is identified as the G′–G″ crossover point

**Fig. 2:**
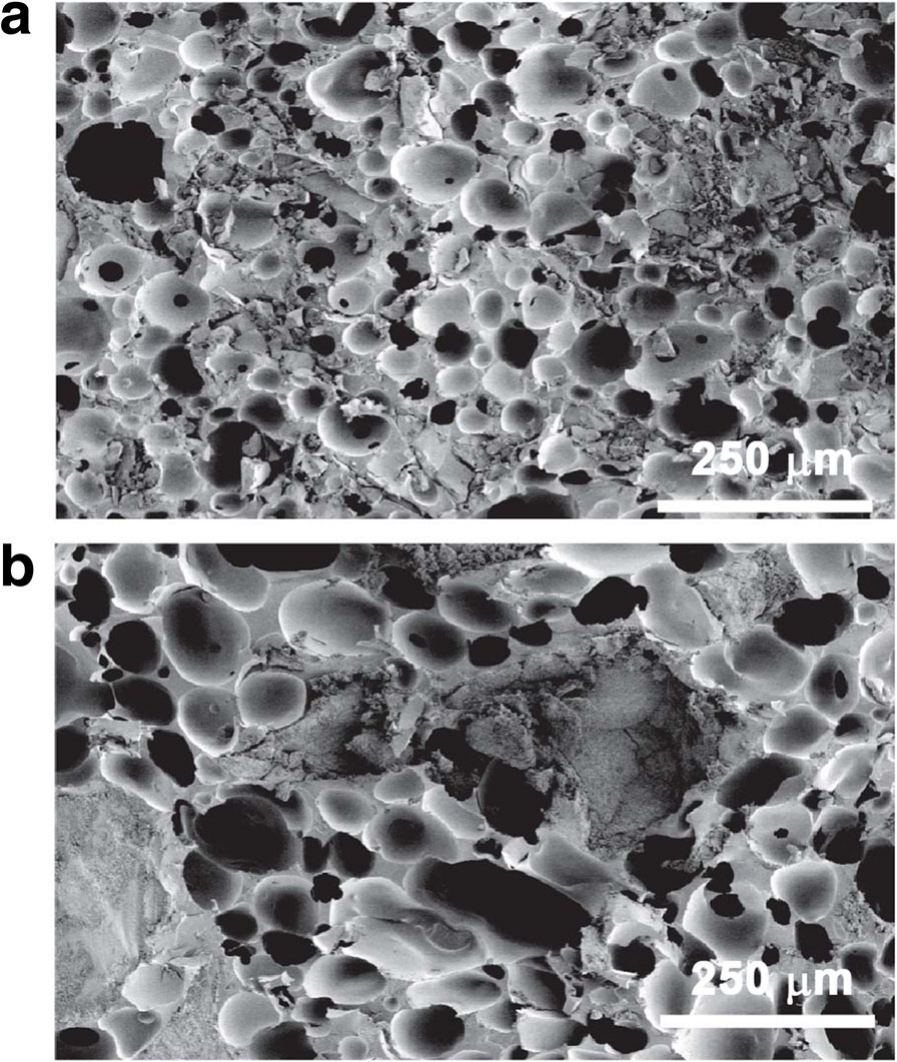
Scanning electron micrographs of **a** LV/BG and **b** LV/CM composites

**Fig. 3:**
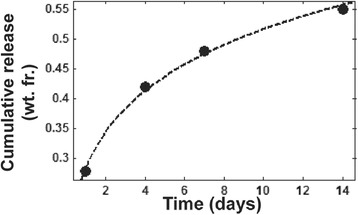
Cumulative fractional release of rhBMP-2 from LV/CM scaffolds measured by ELISA

The LV/BG composite exhibited rheological properties optimal for injectable/settable materials. It expressed shear-thinning properties, with an initial viscosity of 65.7 ± 4.1 Pa s and a working time of 11.5 ± 0.3 min, as determined by the crossover of the storage and loss moduli (Fig. 1a, b). It was not possible to test LV/CM composites due to the larger size of the particles (100–500 μm) and granularity of the mixture. The TFT of LV/BG and LV/CM were 10.3 ± 0.8 and 9.6 ± 0.5 min. The porosity was similar for the two ceramic composite groups (Table 2); however, SEM analysis revealed a modest, but significant, increase in pore size for the LV/CM composites (Table 2, Fig. 2a, b). Interestingly, despite the larger pore size, the LV/CM had a higher bulk moduli at 3.2 MPa than the LV/BG at 1.2 MPa. rhBMP-2 continued to release from LV/CM steadily over a 14-day period (Fig. 3). Approximately 55% of the rhBMP-2 was released after 2 weeks.

**Table 2:** Characteristics and mechanical properties of LV-PUR/ceramic composite bone grafts

Treatment group	Pore size (μm)	Porosity (vol%)	Bulk modulus (MPa)	Yield strength (MPa)
LV/BG	89 ± 2	52.4 ± 0.3	1.2 ± 0.1	0.37 ± 0.03
LV/CM	100 ± 1*	48.0 ± 3.0	3.1 ± 0.4*	0.38 ± 0.05

Data are shown as mean ± SD

*Statistical significance (*p* = 0.05)

### Posterolateral fusion model

**Fig. 4:**
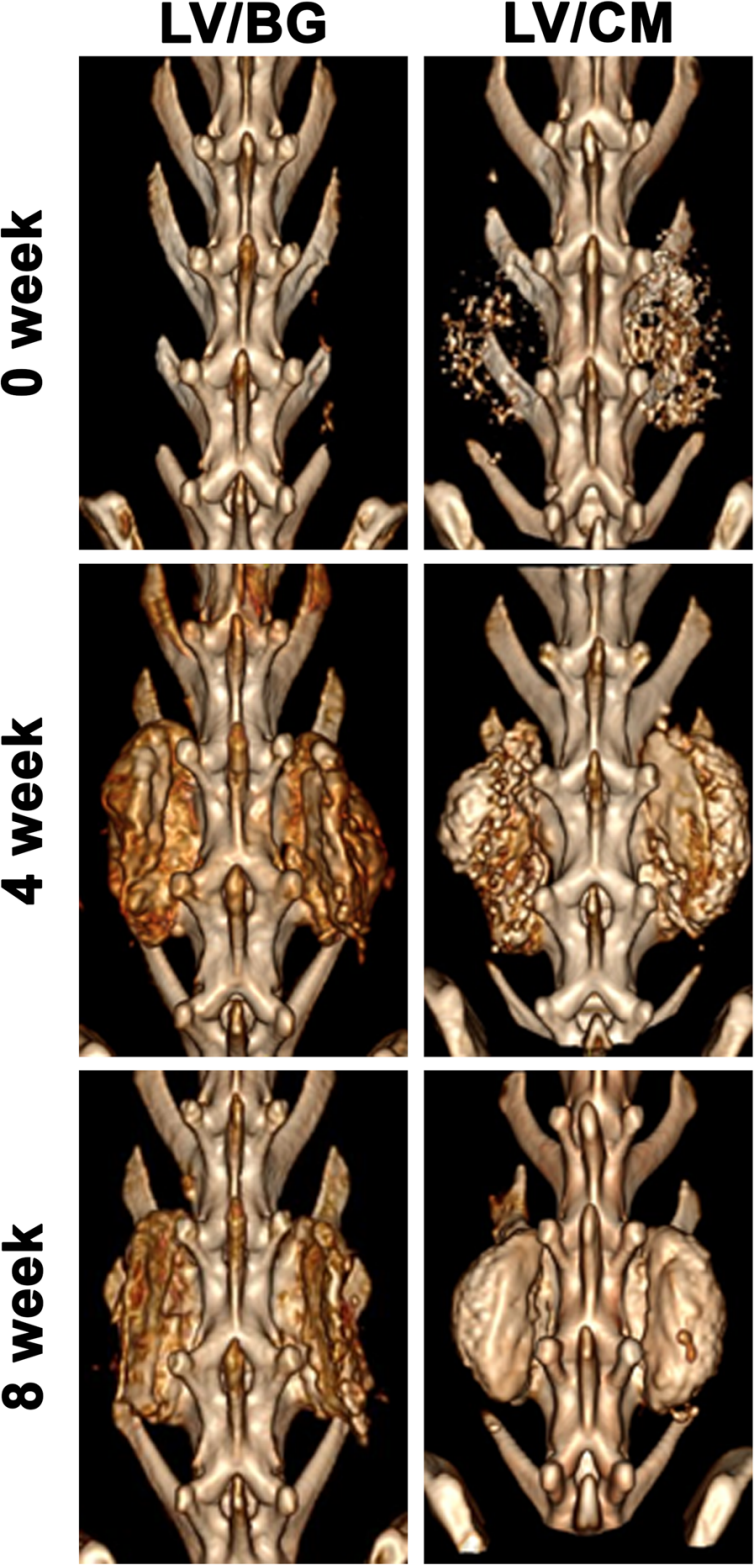
Representative 3D renderings of CT scans taken at 0 (post-operative), 4, and 8 weeks after implantation of the bone graft at the fusion site for LV/BG and LV/CM ceramic composites

**Fig. 5:**
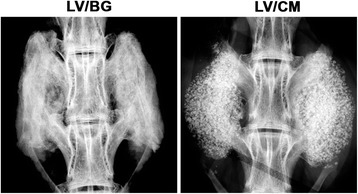
Representative plain radiographs of rabbit spinal fusion sites at 8 weeks for LV/BG and LV/CM ceramic composites

**Fig. 6:**
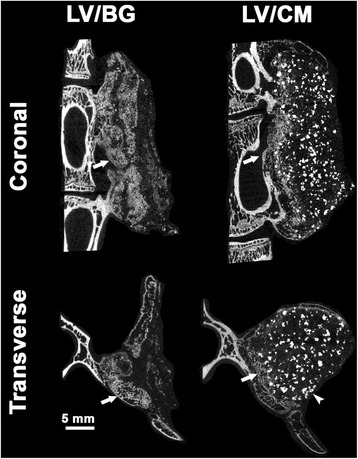
Representative μCT images of LV/BG and LV/CM ceramic composites in the coronal and transverse planes. New bone formation (*arrow*) is noted adjacent to the vertebral body. While the BG particles are indistinguishable from new bone, residual CM appear as bright spots (*arrow head*) in both coronal and transverse view (*scale bar* = 5 mm)

Complete fusion was measured in all animals (3/3) in both treatment groups as determined by manual palpation of the fusion body immediately after the samples were explanted. All samples showed a visual and progressive increase in bone volume over the 8-week study (Fig. 4). The CM component is radiopaque, therefore visible in the initial, 0 week CT. Using plain radiography, new bone formation is evident in the LV/BG group with substantial bone formation on both sides of the fusion site (Fig. 5). Residual CM is visible as bright white particles of the LV/CM composites. μCT images also indicated new bone formation in both the LV/BG and LV/CM groups between the transverse processes, as well as expansion of the composites outside the paraspinal bed (Fig. 6). As with plain radiography, residual CM is visible as bright white particles in the μCT images. Taken together, these radiographic images provide evidence of bone formation and spinal fusion between L5 and L6, which is consistent with the manual palpation data.

**Fig. 7:**
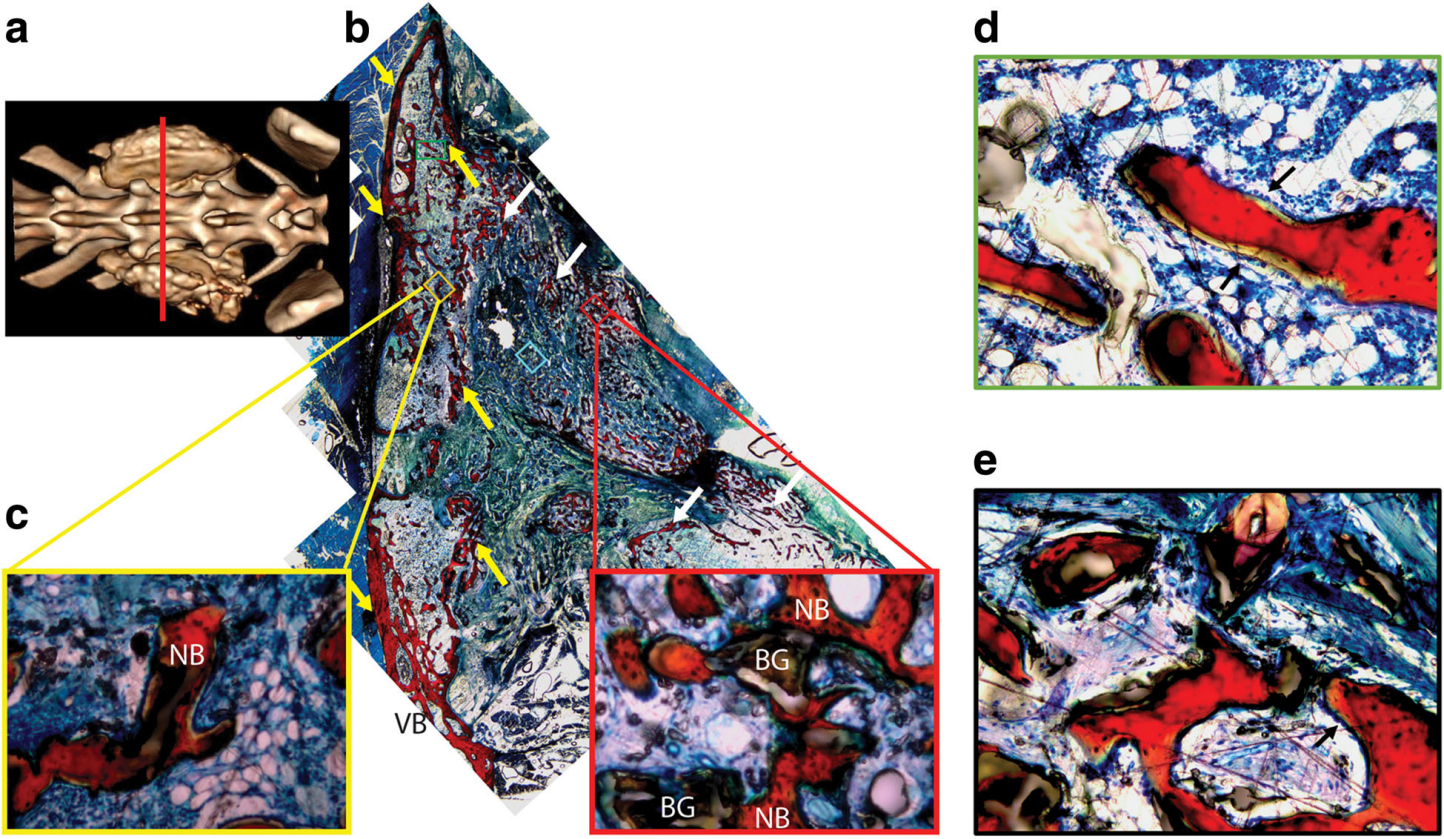
Representative histological sections of LV/BG composites in the transverse plane. **a** CT image of LV/BG composite. The *red line* indicates the location of the coronal section in the mid-section between L5 and L6. **b** Low (×2)-magnification image of a transverse histological section. *White arrows* point to new bone formed outside the fusion body, and *yellow arrows* point to the new bone formed as part of the fusion body between the two transverse processes at the mid-section. Cells are stained *blue*, bone is stained *red*, fibrous tissue is stained *green*. **c** High (×20)-magnification images showing new bone formed in the fusion body. *NB* denotes new bone, and *BG* represents residual bioactive glass particles. **d**, **e** High (×20)-magnification images of bone growth **d** within and **e** away from the fusion mass. *Arrows* point to bone lining, osteoblast-like cells

**Fig. 8:**
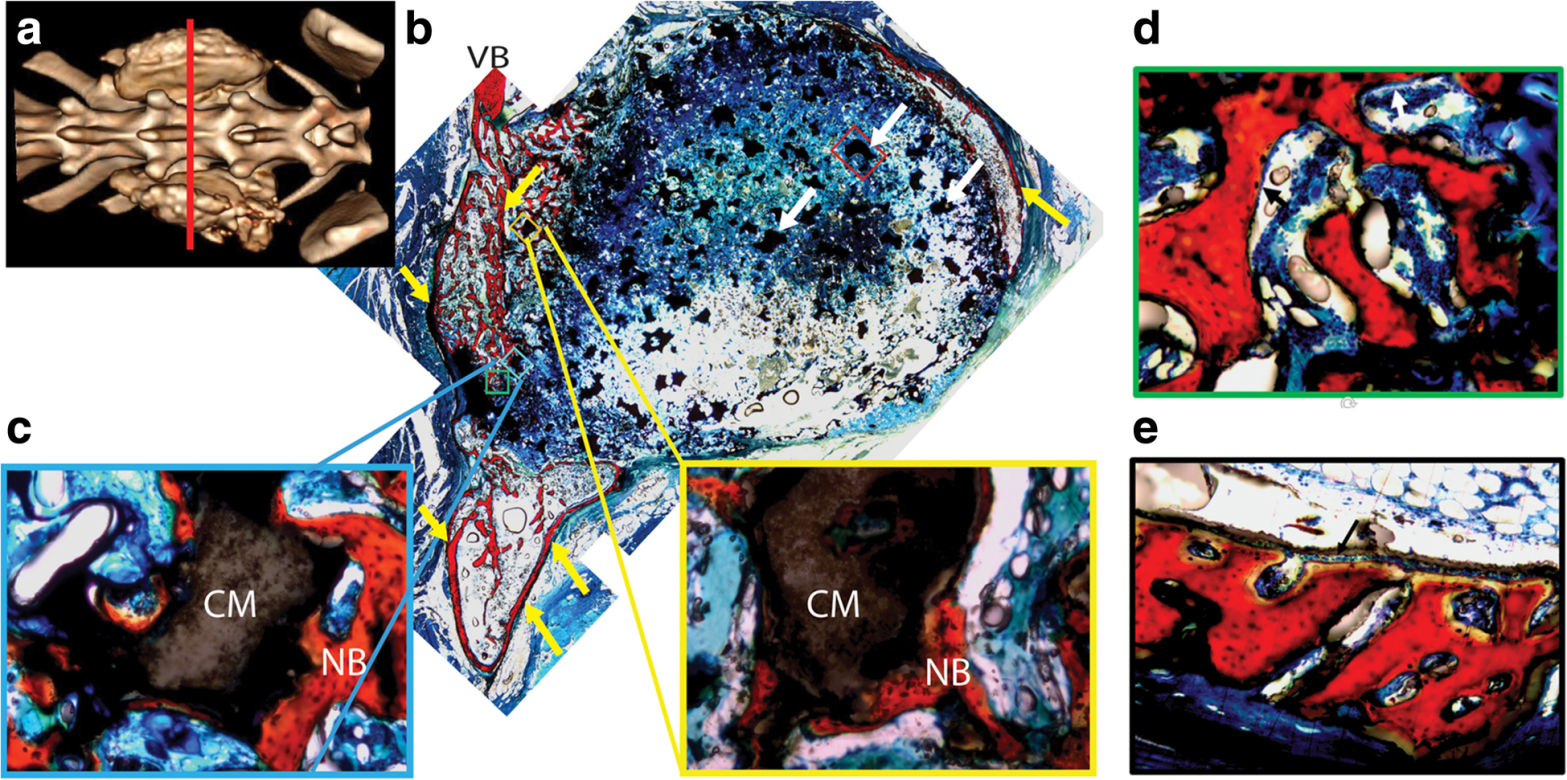
Representative histological sections of LV/CM composites in the transverse plane. **a** CT image of LV/CM composite. The *red line* indicates the location of the coronal section in the mid-section between L5 and L6. **b** Low (×2)-magnification image of a transverse histological section. *White arrows* point to residual CM particles, and *yellow arrows* point to the new bone formed as part of the fusion body between the two transverse processes at the mid-section. Cells are stained *blue*, residual MG is *black*, bone is *red*, and fibrous tissue is *green*. **c** High (×20)-magnification images showing new bone formation on the surface of the CM particles. *NB* denotes new bone, and *CM* represents residual ceramic particles. **d**, **e** High (×20)-magnification images of bone growth **d** within and **e** away from the fusion mass. *Arrows* point to bone lining, osteoblast-like cells

Histology confirms bone formation within the fusion sites from both the LV/BG (Fig. 7) and the LV/CM (Fig. 8) composites. New bone (red) has forms adjacent to the vertebral body, spanning the distance between the transverse processes. There is more evidence of host infiltration, cells, and fibrous tissue, when LV/BG was implanted compared to the LV/CM, coinciding with visually inspected smaller quantities of the ceramic component in the LV/BG group. While there is less evidence of infiltration and ceramic resorption in the LV/CM group, as indicated by the numerous opaque areas, there is evidence of new bone formation at the graft/host interface. Also, the LV/CM group maintained the original graft space whereas the fast LV/BG group reduced in size, indicating quicker graft resorption. No significant difference in total bone area was observed between the LV/BG and LV/CM groups.

## Discussion

LV-PUR/ceramic composites augmented with 430 μg/mL rhBMP-2 exhibited compression resistance, supported diffusion-controlled release of rhBMP-2, and promoted new bone formation and spinal fusion in a PLF in a rabbit model. The properties of the LV-PUR/ceramic composites were favorable for an injectable application, such as MIS, by maintaining low viscosity for close to 10 min. Upon setting, the composites expressed compression resistance with bulk moduli greater than 1 MPa, thus predicted to withstand the compressive forces of the local musculature. Three modes of measurement confirmed fusion in all animals of both groups, and this, while a small group size, can be interpreted positively [13].

An injectable, settable, and compression-resistant carrier for rhBMP-2 administered by MIS techniques has been postulated to decrease complications and length of hospital stays [27]. While injectable and compression-resistant carriers have been reported to maintain space and prevent soft tissue prolapse in a canine mandibular ridge model [16], these carriers have not been evaluated in a more stringent preclinical PLF model. Thus, the aim of this pilot study was to introduce and evaluate injectable, settable, and compression-resistant LV-PUR ceramic carriers for rhBMP-2 for spinal arthrodesis. Ideally, an rhBMP-2 bone graft substitute for MIS applications for PLF should have several characteristics: low viscosity for injectability, a reasonable working time, a compression-resistant matrix, steady rhBMP-2 release, and a resorption rate that matches new bone formation. Several products, both in the market and under investigation, have addressed a number but not all these characteristics. Gold standard rhBMP-2 delivery for lumbar arthrodesis is via an ACS. While the ACS carrier retains rhBMP-2 at the site of placement for 2 weeks [28], the ACS carrier is ineffective for PLF at clinically relevant doses due to soft tissue compression of the collagen [29]. A compression-resistant collagen/ceramic (85/15 β-TCP/HA) matrix (CRM) augmented with 430 μg/mL rhBMP-2 resulted in successful fusion in a rabbit arthrodesis modal, as determined by manual palpation and radiography [30]. This CRM carrier also promoted fusion in non-human primate models of PLF [8, 9], and patients treated with the CRM carrier exhibited superior fusion at 1 year as compared to patients treated with autograft [10]. The relatively high [31] bulk modulus of the CRM resisted the compressive forces of the musculature, resulting in enhanced fusion. These studies also demonstrated that that fusion mass could be augmented by increasing the volume of the carrier and identified the concentration of rhBMP-2 defined on the basis of carrier volume as a critical variable [10]. However, the CRM matrix is not injectable and therefore cannot be combined with MIS surgical techniques. In the present study, LV grafts augmented with the recommended concentration for the ACS carrier in rabbits (430 μg rhBMP-2/mL defined on graft volume) supported spinal fusion in a pilot study of rabbit PLF. These findings are consistent with a previous study that reported that LV bone grafts augmented with 400 μg rhBMP-2/mL supported new bone formation and preserved the anatomy of the mandibular ridge in a canine model [16]. While space maintenance cannot be assessed in the rabbit PLF model, the relatively high bulk modulus of LV-PUR grafts, which is comparable to that of the CRM, suggests that these carriers would be compression-resistant in primates and humans.

This pilot study was designed to answer the question whether injectable, settable, and compression-resistant low-viscosity poly(ester urethane)/ceramic composite grafts augmented with rhBMP-2 could promote fusion in a single-level rabbit PLF model. No differences in fusion rates (100%) or new bone area were observed between groups, which may be due in part to the small sample size (*n* = 3 per group). When used in this model, autograft has a fusion rate of 66% and rhBMP-2-loaded ACS has a fusion rate of 100% but at increased concentrations [7, 25]. Additional dose-response studies with larger sample sizes and clinical controls are needed to establish that the LV-PUR/ceramic composite carriers are equivalent to the ACS carrier at comparable doses of rhBMP-2. The favorable combination of injectability, settability, compression-resistant mechanical properties, and sustained release of rhBMP-2 highlights the potential of LV-PUR/ceramic composite carriers for spinal fusion procedures using MIS techniques.

## Conclusion

In this study, we investigated injectable and settable LV polymer/composite bone grafts as injectable, compression-resistant carriers for rhBMP-2 in a single-level rabbit PLF model. Successful fusion was achieved in animals treated with either LV/CM or LV/BG composites, as evidenced by manual palpation. Images of μCT and histological sections revealed evidence of bone fusion near the TPs. This study highlights the potential of LV grafts augmented with rhBMP-2 as injectable bone grafts for MIS posterolateral spinal fusion surgeries.

## Abbreviations

ACS: Absorbable collagen sponge
BG: Bioglass
CM: Ceramic matrix (85% β-tricalcium phosphate/15% hydroxyapatite (85%βTCP/15%HA))
CRM: Compression-resistant matrix
CT: Computed tomography
G′: Storage modulus
G″: Loss modulus
LTI-PEG: Lysine-triisocyanate polyethylene glycol
LV: Low viscosity
LV/BG: Low-viscosity poly(ester urethane) and bioglass composite
LV/CM: Low-viscosity poly(ester urethane) and ceramic matrix composite
LV-PUR: Low-viscosity poly(ester urethane)
MIS: Minimally invasive surgery
PLF: Posterolateral fusion
rhBMP-2: Recombinant human bone morphogenetic protein-2
TEDA: Triethylene diamine
μCT: Microcomputed tomography
